# Identification and antimicrobial resistance prevalence of pathogenic *Escherichia coli* strains from treated wastewater effluents in Eastern Cape, South Africa

**DOI:** 10.1002/mbo3.319

**Published:** 2016-01-13

**Authors:** Martins A. Adefisoye, Anthony I. Okoh

**Affiliations:** ^1^ SAMRC Microbial Water Quality Monitoring CentreUniversity of Fort HareAlice5700South Africa; ^2^ Applied and Environmental Microbiology Research GroupDepartment of Biochemistry and MicrobiologyUniversity of Fort HareAlice5700South Africa

**Keywords:** Antibiotic‐resistance gene, *E. coli*, MARI, MARP, multidrug resistance, public health

## Abstract

Antimicrobial resistance (AMR) is a global problem impeding the effective prevention/treatment of an ever‐growing array of infections caused by pathogens; a huge challenge threatening the achievements of modern medicine. In this paper, we report the occurrence of multidrug resistance (MDR) in *Escherichia coli* strains isolated from discharged final effluents of two wastewater treatment facilities in the Eastern Cape Province of South Africa. Standard disk diffusion method was employed to determine the antibiotic susceptibility profile of 223 polymerase chain reaction (PCR)‐confirmed *E*. *coli* isolates against 17 common antibiotics in human therapy and veterinary medicine. Seven virulence associated and fourteen antibiotic resistance genes were also evaluated by molecular methods. Molecular characterization revealed five pathotypes of *E. coli* in the following proportions: enterotoxigenic ETEC (1.4%), enteropathogenic EPEC (7.6%), enteroaggregative EAEC (7.6%), neonatal meningitis (NMEC) (14.8%), uropathogenic (41.7%), and others (26.9%). Isolates showed varying (1.7–70.6%) degrees of resistance to 15 of the test antibiotics. Multidrug resistance was exhibited by 32.7% of the isolates, with the commonest multiple antibiotic‐resistant phenotype (MARP) being AP‐T‐CFX (12 isolates), while multiple antibiotic‐resistant indices (MARI) estimated are 0.23 (Site 1) and 0.24 (Site 2). Associated antibiotic resistance genes detected in the isolates include: *str*A (88.2%), *aad*A (52.9%), *cat* I (15%), *cmlA*1 (4.6%), *bla* TEM (56.4%), *tet*A (30.4%), *tet*B (28.4%), *tet*C (42.2%), *tet*D (50%), *tet*K (11.8%), and *tet*M (68.6%). We conclude that municipal wastewater effluents are important reservoirs for the dissemination of potentially pathogenic *E. coli* (and possibly other pathogens) and antibiotic resistance genes in the aquatic milieu of the Eastern Cape and a risk to public health.

## Introduction

Antimicrobial resistance (AMR) is a global health concern responsible for rising incidences of both debilitating and lethal diseases (WHO 2015). Understanding the mechanisms by which microorganisms obtain and share AMR can assist in the development of new interventions to curb this phenomenon (Ahmed et al. 2010). Increasing development of resistance to established antibiotics has taken a center stage in prophylactic and curative medicine worldwide, and more importantly in low‐income African countries (Ndihokubwayo et al. 2013). The proliferation of antibiotic resistance is currently outpacing the development of novel antibiotics (Fahrenfeld et al. 2013), and the rising resistance in bacteria has been reported mainly due to mobile genetic elements that can be readily spread through bacterial populations (Kumarasamy et al. 2010). Changes in bacteria genome can either be by de novo mutation or via horizontal acquisition of resistant genetic materials, which expands the genome of such bacteria, and causing mutation by altering the pre‐existing DNA of the cell (Bennett 2008). Health problems associated with antibiotic‐resistant microorganism is less of disease pathology, but more of limited therapeutic remedies, particularly in much of the developing countries lacking access to good quality treatments, and consequently, infections continue to be an important cause of morbidity and mortality (Samie et al. 2012).

Antibiotic resistance genes have been detected and quantified from different environmentally relevant matrices, including treated wastewater effluents, which are known to contribute to resistant gene loading of surface waters (Fahrenfeld et al. 2013). Wastewater reclamation facilities have been recognized as reservoirs for antibiotic resistance genes associated with human and animal pathogens (Rizzo et al. 2013; Rahube et al. 2014). Antibiotic‐resistant bacteria and unabsorbed antibiotic residues are excreted in urine and feces, and ultimately travel to wastewater reclamation facilities via domestic sewer lines (Rizzo et al. 2012). Classes of antibiotic residues that have frequently been detected in reclaimed municipal effluents include *β*‐lactam, macrolides, lincosamide, tetracyclines, sulphonamides, and fluoroquinolones (Rahube et al. 2014).

Environmental bacteria communities, including *E. coli* has been associated with recognized antibiotic‐resistant gene pools that have been transferred into normal human and animal flora, where they exert strong selective pressure for the emergence and spread of resistance (Kinge et al. 2010; Alves et al. 2014). Even though, most *E. coli* are commensal member of the normal intestinal flora, some virulent strains of the bacteria can cause gastrointestinal infections along with other serious extraintestinal health complications (Stecher and Hardt 2008; Katouli 2010). *Escherichia coli* is classified as either intestinal pathogenic *E. coli* (InPEC) or extraintestinal pathogenic *E. coli* (ExPEC). Intestinal pathogenic *E. coli* (InPEC) strains can cause different forms of gastroenteritis, and are classified into six groups. These are enterohemorrhagic (EHEC), enteropathogenic (EPEC), enteroaggregative (EAEC), enterotoxigenic (ETEC), enteroinvasive (EIEC), and diffusely adherent (DAEC) *E. coli*, while ExPEC (uropathogenic *E. coli* and neonatal meningitis *E. coli*) are causative agents of infections in anatomical sites outside of the gastrointestinal tract, and are associated with urinary tract infections, neonatal meningitis, and septicemia (Lamprecht et al. 2014). *Escherichia coli* can be used as surrogate for antibiotic resistance surveillance because it is found more commonly in diverse hosts and environments, it acquires resistance easily (Erb et al. 2007), and is a reliable indicator of resistance in other pathogenic bacteria such as *Salmonellae* (McEgan et al. 2013; Nsofor et al. 2013).

Nonetheless, numerous studies on the multidrug resistance (MDR) profiling of bacteria have focused mostly on isolates from clinical and food sources, with little information available on the MDR profiles of potentially pathogenic bacteria from final effluents in South Africa. Considering the importance of wastewater effluents as hotspot or potential reservoirs for the dissemination of pathogens and antibiotic resistance genes in the environment hence, the need for such information becomes imperative. Consequently, this study aimed at, investigating the prevalence and antimicrobial resistance patterns of pathogenic *E. coli* strains isolated from treated final effluents in the Eastern Cape, South Africa.

## Materials and Methods

### Study design and source of samples

Wastewater final effluent samples were collected monthly; two samples per site (a total of 48 samples), over a 12 month sampling period (September 2012–August 2013) from two wastewater treatment facilities in Amathole District Municipality, Eastern Cape, South Africa. The two facilities are located within the geographical coordinates 32°34′17′′S, 27°26′95′′E (Site 1), and 32º41′31′′S, 27º08′36′′E (Site 2), respectively. Both treatment plants use the activated sludge and drying beds technology, and disinfect their final effluents by chlorination before discharging into the receiving watersheds.

### Isolation, identification, and molecular characterization of *E. coli* isolates

The samples collected were analyzed by standard membrane filtration technique according to (APHA/AWWA/WEF 1998). Three hundred presumptive *E. coli* isolates were recovered on *E. coli*‐Coliforms Chromogenic medium (Laboratorios CONDA, South Africa) incubated at 37°C for 24 h. About 5–7 randomly selected distinct blue colonies were isolated per plate and subjected to preliminary identification by Gram staining and oxidase testing. Further identification of the selected colonies was done by polymerase chain reaction (PCR). Bacterial DNA was extracted by boiling method as described by Queipo‐Ortuno et al. (2008) with modifications. The isolates were confirmed using housekeeping *uidA* [*β*‐D glucuronidase] gene as described by Janezic et al. (2013). Polymerase chain reaction (PCR)‐confirmed *E. coli* isolates were then stored in 20% glycerol and frozen at −80°C until further analysis.

The confirmed isolates were delineated by PCR into different *E. coli* pathogenic strains or pathotypes based on the presence of virulence genes in their genome according Vidal et al. (2005). The pathotypes assayed and the virulence genes used for their characterization included EPEC, *eae* (Stanilova et al. 2011); ETEC, *lt* and *st* (Stacy‐Phipps et al. 1995; Vidal et al. 2005); EAEC, *eagg* (Pass et al. 2000); EIEC, *ipaH* (Vidal et al. 2005); DAEC, *daaE* (Vidal et al. 2005); UPEC, *papC* (Hilali et al. 2000) and NMEC, *ibeA* (Cebula et al. 1995). *Escherichia coli* DSM reference strains (DSM 8695, DSM 10 973, DSM 10 974, DSM 4816, DSM 10 819, DSM 9025) were included in the assays as internal positive controls for the targeted *E. coli* pathotypes, while the negative control consisted of the PCR buffer and nuclease‐free water.

### Antibiotic resistance profiling of *E. coli* strains

The antibiotic resistance/susceptibility profiles of the PCR‐confirmed isolates were determined by disk diffusion test (CLSI, 2011). Seventeen commercial antibiotic discs (Mast Diagnostics, Merseyside, UK) which include: ampicillin, amikacin, imipenem, meropenem, streptomycin, chloramphenicol, ciprofloxacin, cephalexin, nalidixic acid, tetracycline, norfloxacin, gentamicin, cefuroxime, cefotaxime, polymyxin B, colistin sulfate, and nitrofurantoin were employed for the susceptibility testing. The profiles of the isolates were determined by measuring the diameters of the zones of inhibition, and comparing them to the Clinical and Laboratory Standards Institute (CLSI) interpretative charts. Multidrug resistance (multiple antibiotic resistance phenotype) was defines as the exhibition of resistance to three or more different classes of antibiotics, while multiple antibiotic resistance indices(MARI) of the isolates were estimated as previously described by Krumperman (1983). MAR index (MARI) = *a*/(*b *× *c*); where *a*, is the aggregate antibiotic resistance score of isolates; *b*, is the number of antibiotics, and; *c*, is the number of isolates.

### Detection of antibiotic‐resistant genes

Polymerase chain reactions with specific oligonucleotide primers were used to test for the occurrence of antibiotic‐resistant genes in the 73 (32.7%) PCR‐confirmed isolates that showed multidrug resistance to the test antibiotics. Fourteen antibiotic resistance genes cutting across different classes of antibiotics were assayed following previously described PCR protocols with some modifications. These included aminoglycosides [*str*A, *aad*A (Velusamy et al. 2007)], phenicols [*cat* I, *cat* II (Maynard et al. 2004), *cml*A1 (Post and Hall 2009)], *β*‐lactam [*amp*C (Velusamy et al. 2007), *bla*Z (Baddour et al. 2007), *bla*TEM (Bailey et al. 2010)], and tetracycline [*tet*A, *tet*B, *tet*C, *tet*D (Ng et al. 2001), *tet*K, *tet*M (Strommenger et al. 2003)].

## Results

### Molecular identification and characterization of *E. coli* isolates

Two hundred and twenty‐three presumptive *E. coli* isolates were identified by Gram staining, oxidase test, and PCR (*uid*A gene, 147 bp). Further molecular characterization of the PCR‐confirmed *E. coli* isolates into different strains based on the detection of the various virulence genes in their genomes revealed different proportions as follows: *eae* 17 (7.6%), *lt* 3 (1.4%), *eagg* 17 (7.6%), *papC* 93 (41.7%), *ibeA* 33 (14.8), other/uncharacterized 60 (26.9%), while *ipaH* and *daaE* genes were not detected in any of the isolates.

### Antibiotic resistance profiling of confirmed isolates

The antibiotic resistance profiles of the confirmed *E. coli* strains are presented in Table 1. All the isolates were susceptible to both meropenem and imipenem, while one isolate was resistant to each of gentamycin and amikacin. Of the test antibiotics, tetracycline is the antibiotic to which the highest frequencies of resistance (60.1%) was shown; this is followed by ampicillin with frequencies of 55.6% and cephalexin 51.1% (Table 1). About 33% of the ETEC strains showed resistance to each of streptomycin, chloramphenicol, cefotaxime, and nitrofurantoin, while 5.9 and 70.6% of the EPEC strains were resistant to ciprofloxacin and tetracycline, respectively. Also, the EAEC strains exhibited resistance ranging between 5.9% and 58.8% to ciprofloxacin, ampicillin, chloramphenicol, tetracycline, nalidixic acid, polymyxin B, colistin sulfate, cephalexin, and nitrofurantoin. For the NMEC strains, 63.6% of the isolates showed resistance against tetracycline, while 54.6% and 33.3% were resistant to ampicillin and nalidixic acid, respectively. UPEC strains showed highest frequency of resistance (63.4%) against tetracycline, while 55.9% and 48.4% of the isolates were resistant to cephalexin and ampicillin, respectively. About 1.7% of uncharacterized pathotypes were resistant to norfloxacin, gentamycin, and amikacin, while 68.3% showed resistance against ampicillin.

**Table 1:** Antibiotic resistance profiles of *Escherichia coli* pathotypes showing percentages of resistance to the test antibiotics

Antibiotic	Resistance profiles (%)						Frequency of resistance (*n *=* *223)
	ETEC (*n *=* *3)	EPEC (*n *=* *17)	EAEC (*n *=* *17)	NMEC (*n *=* *33)	UPEC (*n *=* *93)	Others (*n *=* *60)	
Amikacin [AK] 30 *μ*g	–	–	–	–	–	1 (1.7%)	1 (0.5%)
Ampicillin [AP] 25 *μ*g	2 (66.7%)	9 (52.9%)	9 (52.9%)	18 (54.6%)	45 (48.4%)	41 (68.3%)	124 (55.6%)
Ceforoxime [CXM] 30 *μ*g	–	–	–	6 (18.2%)	4 (4.3%)	4 (6.7%)	14 (6.3%)
Cefotaxime [CTX] 30 *μ*g	1 (33.3%)	1 (5.9%)	–	–	6 (6.5%)	2 (3.3%)	10 (4.5%)
Cephalexin [CFX]	2 (66.7%)	7 (41.2%)	8 (47.1%)	–	52 (55.9%)	45 (75%)	114 (51.1%)
Chloramphenicol [C] 10 *μ*g	1 (33.3%)	3 (17.7%)	1 (5.9%)	8 (24.2%)	13 (14.0%)	25 (41.7%)	51 (22.9%)
Ciprofloxacin [CIP] 5 *μ*g	–	1 (5.9%)	2 (11.8%)	4 (12.1%)	2 (2.2%)	–	9 (4%)
Colistin sulfate [CO] 10 *μ*g	–	1 (5.9%)	1 (5.9%)	–	7 (7.5%)	5 (8.3%)	14 (6.3%)
Gentamycin [GM] 120 *μ*g	–	–	–	–	–	1 (1.7%)	1 (0.5%)
Imipenem [IMI] 10 *μ*g	–	–	–	–	–	–	–
Meropenem [MEM] 10 *μ*g	–	–	–	–	–	–	–
Nalidixic [NA] 30 *μ*g	–	5 (29.4%)	7 (41.2%)	11 (33.3%)	21 (22.6%)	26 (43.3%)	70 (31.4%)
Nitrofurantoin [NI] 300 units	1 (33.3%)	–	2 (11.8%)	2 (6.1%)	1 (1.1%)	6 (10%)	12 (5.4%)
Norfloxacin [NOR] 10 *μ*g	–	1 (5.9%)	–	6 (18.2%)	3 (3.2%)	1 (1.7%)	11 (4.9%)
Polymyxin B [PB] 300 units	–	3 (17.6%)	1 (5.9%)	–	5 (5.4%)	3 (5%)	12 (5.4%)
Streptomycin [S] 10 *μ*g	1 (33.3%)	3 (17.7%)	–	5 (15.2%)	10 (10.8%)	–	18 (8.1%)
Tetracycline [T] 30 *μ*g	2 (66.7%)	12 (70.6%)	10 (58.8%)	21 (63.6%)	59 (63.4%)	30 (50%)	134 (60.1%)

Two different multiple antibiotic‐resistant phenotypes (MARP) that is, AP‐T‐CFX‐NI and S‐AP‐CXM‐T‐CTX‐CFX were observed among the ETEC strains, both at a frequency of 33.3% (1/3). Similarly, seven different MARP were observed among the EPEC strains, at a frequency of 5.9% (1/17). The predominant MARP observed among the EAEC strains was AP‐T‐NA‐CFX at 17.7% (3/17), while S‐CIP‐AP‐NOR‐T‐NA (9.1%) was the mostly observed MARP for NMEC strains. For UPEC pathotype, AP‐T‐CFX was the most prevalent (7.5%) MARP. For the uncharacterized *E. coli* isolates, MARP AP‐C‐NA‐PB was the most predominant, occurring at a frequency of 8.3% (5/6). The multiple antibiotic resistance indices (MARI) estimated for the two sampling sites are 0.23 (site 1) and 0.24 (site 2).

### Detection of antibiotic resistance genes

**Figure 1:**
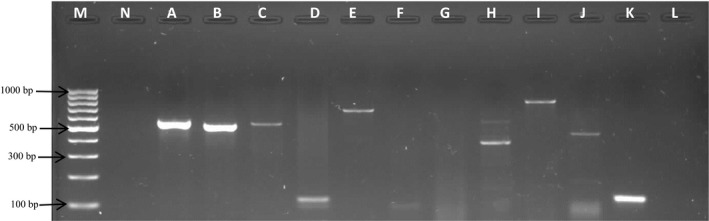
Antibiotic‐resistant determinants. Lane M: 100 bp molecular weight marker; lane N: negative control; lane A: *str*A (546 bp); lane B: *aad*A (525 bp); lane C: *cat* I (547 bp); lane D: *cmlA*1 (115 bp); lane E: *bla* TEM (690 bp); lane H: *tet*C (385 bp); lane I: *tet*D (787 bp); lane J: *tet*K (460 bp); lane K: *tet*M (125 bp).

Molecular detection of antibiotic resistance genes in the multidrug‐resistant isolates revealed the presence of 11 different resistant genes. The *cat* II, *amp*C, and *bla*Z genes were not detected in any of the isolates, even though, some isolates showed resistance to these classes of antibiotic. The distribution of the antibiotic resistance genes in the various resistance strains is shown in Table 2, and generally ranged between 4.6% (*cmlA*1) and 88.2% (*str*A). Some isolates were found to harbor antibiotic resistance genes for two or more classes of antibiotics. For instance, six isolates belonging to different pathotypes carried *str*A and *aad*A (both conferring resistance to aminoglycosides) as well as *bla*TEM (conferring resistance to *β*‐Lactam). Figure 1 below shows a representative gel electrophoretic image of some of the detected antibiotic resistance genes.

**Table 2:** Distribution of antibiotic‐resistant genes *Escherichia coli* isolates

Class of antibiotic	Antibiotic‐resistant genes	Number (%) of positive isolates
Aminoglycosides	*strA*	15 (88.2)
	*aadA*	9 (52.9)
Phenicols	*cat I*	7 (15.9)
	*cat II*	0
	*cmlA*1	2 (4.6)
*β*‐Lactam	*ampC*	0
	*blaZ*	0
	*bla*TEM	53 (56.4)
Tetracycline	*tet*A	31 (30.4)
	*tet*B	29 (28.4)
	*tet*C	43 (42.2)
	*tet*D	51 (50)
	*tet*K	12 (11.8)
	*tet*M	70 (68.6)

## Discussion

Due to its ability to persist in the environment for a considerable period, *E. coli* is used as indicator to predict the possible presence of other bacteria/pathogens particularly those of enteric origins. A study carried out on surface water in Australia suggested that more than 50% of *E. coli* isolates in surface water environment are likely to have originated from wastewater effluents (Anastasi et al. 2012), underlining the importance of municipal wastewater treatment plants as potential point sources of pathogens into surface water.

In this study, five different groups of pathogenic *E. coli* belonging to the two broad categories that is, InPEC (ETEC, EPEC and EAEC) and ExPEC (NMEC and UPEC) with frequencies of detection generally ranging between 1.4% and 41.7% were isolated from discharged final effluents of two wastewater treatment facilities in the Eastern Cape Province of South Africa. InPEC strains of *E. coli* also known as diarrheagenic *E. coli* (DEC) are major etiological agents of pediatric diarrhea, which continues to be the most common cause of infantile morbidity and mortality most especially in developing countries. Diarrheagenic *E. coli* (DEC) can be transmitted via the oral‐fecal route by ingesting food or water contaminated by human or animal feces. Pathogenesis of ETEC involves the establishment of adherence to the epithelium of the small intestine by means of colonization factors (CF) (Oh et al. 2014). This is followed by the production of enterotoxins (a heat‐stable toxin (st) and/or a heat‐labile toxin (lt)), which stimulate the lining of the intestine causing secretion of excessive fluid, often leading to diarrhea (CDC 2014). Enterotoxigenic (ETEC) strains are responsible for millions of infections cases worldwide, and it is one of the most important pathogens associated with death following moderate to severe diarrhea in children (Luo et al. 2014). Clinical symptoms of ETEC infection include profuse watery diarrhea with abdominal cramps (usually self‐limiting) while fever, headache, nausea, and vomiting are less common symptoms (Yoder et al. 2006). The nondetection of the *st* gene as observed in our study might have resulted from the loss of the plasmid‐encoded gene (Taxt et al. 2010).

Enteropathogenic (EPEC) strains are classified as either typical or atypical, and are described as attaching and effacing pathogens due to their ability to form distinctive lesions on the surfaces of intestinal epithelial cells. Typical EPEC strains have been recognized as important agents of diarrhea in developing countries while atypical strains have been commonly isolated in developed countries (Santona et al. 2013). These two subgroups of EPEC are differentiated based on the presence or absence of a bundle forming pili gene (*bfpA*) (Santona et al. 2013). Clinical presentation of EPEC infection includes watery diarrhea often accompanied by fever, vomiting, and dehydration in infants. EAEC causes acute or persistent diarrhea among infants, and has been responsible for large outbreaks in Europe, the United Kingdom, Switzerland, and Japan as well as in developing countries (Kaur et al. 2010). Enteroaggregative (EAEC) adheres to the epithelial cells in a distinctive “stacked‐brick” pattern and can form biofilms (Weintraub 2007). The overall pathogenesis mechanisms of this pathogen remain unclear (Arenas‐Hernandez et al. 2012). Some manifestations of infection by EAEC include watery diarrhea, abdominal pain, nausea, vomiting, and low‐grade fever (Vaishnavi 2013).

One of the most relevant diseases caused by ExPEC in animals is systemic colibacillosis leading to significant economic losses in the poultry industry (Ewers et al. 2007). NMEC are the second most common cause of neonatal bacterial meningitis with mortality rate greater than 10% and are responsible for about 20–50% cases of sequelae (Gaschignard et al. 2012). UPEC is involved in a large number of urinary tract infections (UTIs) and infectious complications, which may lead to acute renal failure in healthy individuals as well as in renal transplant patients (Bien et al. 2012). Urinary tract infections (UTIs) are considered the most common infections in humans and are classified into disease categories according to the site of infection (Bien et al. 2012).

Extraintestinal pathogenic *E. coli* (ExPEC) are the predominant strains detected in this study, with frequency of detection ranging between 14.8% and 41.7%. They accounting for 56.5% of the total isolates while InPEC strains (1.4–7.6%) accounts for 16.6% of the isolates. Uncharacterized strains made up 26.9% of the total isolates. Higher prevalence of ExPEC compared to InPEC as recorded in this study is corroborated by other different studies of municipal wastewater effluents (Anastasi et al. 2010; Mokracka et al. 2011; Frigon et al. 2013). In their report, Anastasi et al. (2010) documented a predominance of ExPEC strains (59.5%) while reporting on the prevalence and persistence of *E. coli* strains in four sewage treatment plants in Australia. They concluded that ExPEC (uropathogenic) strains can survive all wastewater treatment processes, thus, increasing their chance of release into surface water and constituting significant public health risk. In a similar study, Mokracka et al. (2011) recorded 50.5% of ExPEC compared to InPEC (21%) strains while investigating the phylogenetics, virulence, and quinolone resistance of integrin‐bearing *E. coli* strains isolated from a wastewater treatment plant. Frigon et al. (2013), also reported an abundance of ExPEC (24%) over InPEC (10%) associated strains while investigating the removal of virulent *E. coli* by biological and physicochemical wastewater treatment processes.

All the confirmed isolates in this study showed marked susceptibility (≥98.3%) to four (meropenem, imipenem, gentamycin and amikacin) of the test antibiotics. This observation is similar to the finding Joly‐Guillou et al. (2010), who reported 100% susceptibility of *E. coli* isolates recovered from the French hospitals meropenem and imipenem, and also with the findings of Rocha et al. (2014), who recorded lack of resistance against imipenem, gentamycin, and amikacin in their study. Based on our observation, these four antibiotics could be very useful as drugs of choice for therapeutic purposes in the event of waterborne outbreaks around the study area. The isolates exhibited varying levels of responses to other classes of antibiotic with tetracycline being the least potent (Table 1), this is followed by ampicillin with resistance frequency of ≥47.8%. Similar findings to our observation have been reported by other studies (Momtaz et al. 2012; Tadesse et al. 2012). Long‐term use of these two classes (tetracycline; introduced in 1948 and ampicillin; introduced in 1961) of antibiotic has been suggested to be responsible for the high level of resistance against them (Tadesse et al. 2012).

Multidrug resistance was observed in 32.7% of the isolates with different multiple antibiotic‐resistant phenotypes (MARP). The predominant MARP was AP‐T‐CFX, found in 12 (5.4%) isolates cutting across different pathotypes. Other MARP showing resistance against 4–7 different antibiotics was also detected at different frequencies. One major implication of multiple antibiotic resistance in *E. coli* and other pathogens is limited treatment options for some bacterial infections that were thought to be curable previously with a huge public health burden. Multidrug resistance has led to the reclassification of certain diseases as re‐emerging with associated health implications such as prolonged illness period, higher cost for therapy, and increased risk of death.

Multiple antibiotic resistance index (MARI) has been used to estimate health risk associated with the spread of drug resistance in an environment. Multiple antibiotic‐resistant indices (MARI) value of 0.2 (arbitrary) is used to differentiate between low‐ and high‐health risk, and MARI greater than 0.2 suggests that a strain(s) of bacteria originate from an environment with high contamination or antibiotics usage (Christopher et al. 2013). The MARI estimates obtained for isolates from out study sites (site 1 (0.23) and site 2 (0.24)) were similar and both greater than 0.2, suggesting that the isolates originated from environments with high use or contamination of antibiotics. The high MARI values obtained in this study may suggest the exposure of the isolates to antibiotics pressure, which might have resulted from inappropriate use of antibiotic among the population in the study area, and may lead further to increase in the development of multidrug resistance overtime if appropriate measures are not put in place.

Molecular analysis of antibiotic resistance gene reveals the presence of 11 genes conferring resistance to different classes of antibiotics. Six different genes conferring resistance against tetracycline were mostly detected at frequencies ranging from 11.8% (*tet* K) to 68.8% (*tet*M), suggesting that resistance to this class of drug may be genetically mediated as a result of long‐term use. Similarly, high detection of *bla*TEM conferring resistance to *β*‐lactam was observed at a frequency of 56.4% and supports the finding of Momtaz et al. (2012), who reported high rate of detection of resistant genes conferring resistance to tetracycline and ampicillin. Some of the isolates in this study were found to harbor multiple resistant genes conferring resistance to two or more different classes of antibiotics. The repertoire of antibiotic‐resistant genes found in the *E. coli* pathotypes might serve as a pointer to the possible presence of other antidrug‐resistant genes conferring resistance to other classes of antibiotics that were not targeted in this study, and our finding is in line with other reports on the detection of multiple antibiotic‐resistance gene in some commensal and pathogenic strains of *E. coli* (Bailey et al. 2010; Karczmarczyk et al. 2011).

## Conclusions

The results obtained in this study clearly showed that the final effluents of wastewater treatment plants are reservoir of antibiotic‐resistant *E. coli* pathotypes (and possibly other pathogens), and potential point sources of antibiotic‐resistant genes, which might be transferred to other pathogens in the receiving watersheds, thus, presenting a public health risk. The combination of the deadly duo of drug resistance and emerging virulence in pathogenic bacteria brings about a worrisome situation of possible lack of therapeutic options for some severe bacterial infections in the nearest future. This phenomenon coupled with high number of immunocompromised individuals in the Southern African region calls for priority attention to bring under control the spread of antibiotic resistance in order to safeguard the health of the general public.

## Conflict of Interest

None declared.

## Supporting information


**Table S1.** Primer sequences, PCR protocols, and sizes of PCR‐amplified target genes of *E. coli* pathotypes.

**Table S2.** Primer sequences and PCR conditions for antibiotic resistant determinants assayed.

**Figure S1.** Gel electrophoresis image of *E. coli* identification. Lane M: 100 bp molecular weight marker (Thermo Scientific Inc.); lane P: positive control (*E. coli* ATCC 25922 strain); lane N: negative control; lanes 1 to 10 PCR‐confirmed *E. coli* isolates.

**Figure S2a.** Molecular detection of NMEC pathotype by the amplification of *ibe*A gene (342 bp). Lane M: 100 bp molecular weight marker (Thermo Scientific Inc.); lane P: positive control (*E. coli* DSM 10819 strain); lane N: negative control; lanes 1 to 10 *E. coli* isolates.

**Figure S2b.** Molecular detection of UPEC pathotype by the amplification of *pap*C gene (382 bp). Lane M: 100 bp molecular weight marker (Thermo Scientific Inc.); lane P: positive control (*E. coli* DSM 4816 strain); lane N: negative control; lanes 1 to 10 *E. coli* isolates.

**Figure S2c.** Molecular detection of EAEC pathotype by the amplification of *eagg* gene (194 bp). Lane M: 100 bp molecular weight marker (Thermo Scientific Inc.); lane P: positive control (*E. coli* DSM 10974 strain); lane N: negative control; lanes 1 to 22 *E. coli* isolates.

**Figure S2d.** Molecular detection of ETEC pathotype by the amplification of *lt* gene (218 bp). Lanes M & Z: 100 bp molecular weight marker (Thermo Scientific Inc.); lane P: positive control (*E. coli* DSM 10973 strain); lane N: negative control; lanes 1 to 9 *E. coli* isolates.

**Figure S2e.** Molecular detection of EPEC pathotype by the amplification of *eae* gene (482 bp). Lanes M & Z: 100 bp molecular weight marker (Thermo Scientific Inc.); lane P: positive control (*E. coli* DSM 10973 strain); lane N: negative control; lanes 1 to 19 *E. coli* isolates.
