# Supplementary material for: Identification and antimicrobial resistance prevalence of pathogenic Escherichia coli strains from treated wastewater effluents in Eastern Cape, South Africa
Source: Microbiologyopen. 2016 Jan 13;5(1):143–51. doi: 10.1002/mbo3.319 (PMC4767426; doi:10.1002/mbo3.319)
Supplement: Supplementary file 1 — Table S1. Primer sequences, PCR protocols, and sizes of PCR‐amplified target genes of E. coli pathotypes. [file MBO3-5-143-s001.pdf]

Supplementary Table 1. Primer sequences, PCR protocols and sizes of PCR-amplified target genes of *E. coli* pathotypes.

**Target Strains**	**Target Genes**	**Primer Sequence (5´→3´)**	**Reaction Conditions (ºC)**	**Cycles**	**Amplicon Size (bp)**	**Positive Control**
EPEC	*eae*	TCAATGCAGTTCCGTTATCAGTT<br>GTAAAGTCCGTTACCCCAACCTG	95º, 94º, 55º, 68º, 72º<br>15´, 45´´, 45´´, 2´, 5´	35	482	DSM 8695
ETEC	*lt*	GCACACGGAGCTCCTCAGTC<br>TCC TTCATCCTTTCAATGGCTTT	95º, 94º, 58º, 72º, 72º<br>3´, 30´´, 1´, 1´, 10´	35	218	DSM 10973
EAEC	*eagg*	AGACTCTGGCGAAAGACTGTATC<br>ATGGCTGTCTGTAATAGATGAGAAC	95º, 94º, 55º, 68º, 72º<br>15´, 45´´, 45´´, 2´, 5´	35	194	DSM 10974
UPEC	*papC*	GACGGCTGTACTGCAGGGTGGCG<br>ATATCCTTTCTGCAGGGATGCAATA	94º, 94º, 55º, 72º, 72º<br>2´, 1´, 1´, 1´, 5´	30	382	DSM 4816
NMEC	*ibeA*	TGGAACCCGCTCGTAATATAC<br>CTGCCTGTTCAAGCATTGCA	95º, 94º, 58º, 72º, 72º<br>3´, 30´´, 1´, 1´, 10´	30	342	DSM 10819
DAEC	*daaE*	GAACGTTGGTTAATGTGGGGTAA<br>TATTCACCGGTCGGTTATCAGT	94º, 92º, 59º, 72º, 72º<br>2´, 30´´,30´´,30´´, 5´	30	542	-
EIEC	*ipaH*	CTC GGCACGTTTTAATAGTCTGG<br>GTGGAGAGCTGAAGTTTCTCTGC	95º, 95º, 55º, 72º, 72º<br>5´, 45´´, 45´´, 45´´, 10´	30	320	DSM 9025
