# Supplementary material for: Identification and antimicrobial resistance prevalence of pathogenic Escherichia coli strains from treated wastewater effluents in Eastern Cape, South Africa
Source: Microbiologyopen. 2016 Jan 13;5(1):143–51. doi: 10.1002/mbo3.319 (PMC4767426; doi:10.1002/mbo3.319)
Supplement: Supplementary file 2 — Table S2. Primer sequences and PCR conditions for antibiotic resistant determinants assayed. [file MBO3-5-143-s002.pdf]

Supplementary Table 2. Primer sequences and PCR conditions for antibiotic resistant determinants assayed.

**Target**	**Primer Sequence**	**Amplicon Size (bp)**	**PCR Condition**	**Cycles**
*str*A	F: CTTGGTGATAACGGCAATTC	546	94º, 94º, 68º, 72º, 72º	30
	R: CCAATCGCAGATAGAAGGC		4´, 45´´, 45´´, 45´´, 7´	
*aad*A	F: GTGGATGGCGGCCTGAAGCC	525	94º, 94º, 68º, 72º, 72º	30
	R: AATGCCCAGTCGGCAGCG		4´, 45´´, 45´´, 45´´, 7´	
*amp*C	F: TTCTATCAAMACTGGCARCC	550	94º, 94º, 60º, 72º, 72º	30
	R: CCYTTTTATGTACCCAYGA		4´, 45´´, 45´´, 45´´, 7´	
cat I	F: AGTTGCTCAATGTACCTATAACC	547	94º, 94º, 50º, 72º, 72º	30
	R: TTGTAATTCATTAAGCATTCTGCC		5´, 30´´, 30´´, 90´´, 5´	
cat II	F: ACACTTTGCCCTTTATCGTC	543	94º, 94º, 50º, 72º, 72º	30
	R: TGAAAGCCATCACATACTGC		5´, 30´´, 30´´, 90´´, 5´	
*cml*A1	F: CACCAATCATGACCAAG	115	94º, 94º, 50º, 72º, 72º	30
	R: GGCATCACTCGGCATGGACATG		5´, 30´´, 30´´, 90´´, 5´	
*bla*Z	F: ACTTCAACACCTGCTGCTTTC	490	94º, 94º, 60º, 72º, 72º	30
	R: TGACCACTTTTATCAGCAACC		5´, 30´´, 30´´, 90´´, 5´	
*bla*TEM	F: TTTCGTGTCGCCCTTATTCC	690	94º, 94º, 60º, 72º, 72º	30
	R: CCGGCTCCAGATTTATCAGC		5´, 30´´, 30´´, 90´´, 5´	
*tet*A	F: GCTACATCCTGCTTGCCTTC	210	94º, 94º, 55º, 72º, 72º	35
	R: CATAGATCGCCGTGAAGAGG		5´, 60´´, 60´´, 90´´, 5´	
*tet*B	F: TTGGTTAGGGGCAAGTTTTG	659	94º, 94º, 55º, 72º, 72º	35
	R: GTAATGGGCCAATAACACCG		5´, 60´´, 60´´, 90´´, 5´	
*tet*C	F: CTTGAGAGCCTTCAACCCAG	418	94º, 94º, 55º, 72º, 72º	35
	R: ATGGTCGTCATCTACCTGCC		5´, 60´´, 60´´, 90´´, 5´	
*tet*D	F: AAACCATTACGGCATTCTGC	787	94º, 94º, 55º, 72º, 72º	35
	R: GACCGGATACACCATCCATC		5´, 60´´, 60´´, 90´´, 5´	
*tet*K	F: GTAGCGACAATAGGTAATAGT	460	94º, 94º, 55º, 72º, 72º	35
	R: GTAGTGACAATAAACCTCCTA		5´, 60´´, 60´´, 90´´, 5´	
tetM	F: AGTGGAGCGATTACAGAA	158	94º, 94º, 55º, 72º, 72º	35
	R: CATATGTCCTGGCGTGTCTA		5´, 60´´, 60´´, 90´´, 5´	
