# Supplementary material for: Identification and antimicrobial resistance prevalence of pathogenic Escherichia coli strains from treated wastewater effluents in Eastern Cape, South Africa
Source: Microbiologyopen. 2016 Jan 13;5(1):143–51. doi: 10.1002/mbo3.319 (PMC4767426; doi:10.1002/mbo3.319)
Supplement: Supplementary file 4 — Figure S2a. Molecular detection of NMEC pathotype by the amplification of ibeA gene (342 bp). Lane M: 100 bp molecular weight marker (Thermo Scientific Inc.); lane P: positive control (E. coli DSM 10819 strain); lane N: negative control; lanes 1 to 10 E. coli isolates. [file MBO3-5-143-s004.pdf]

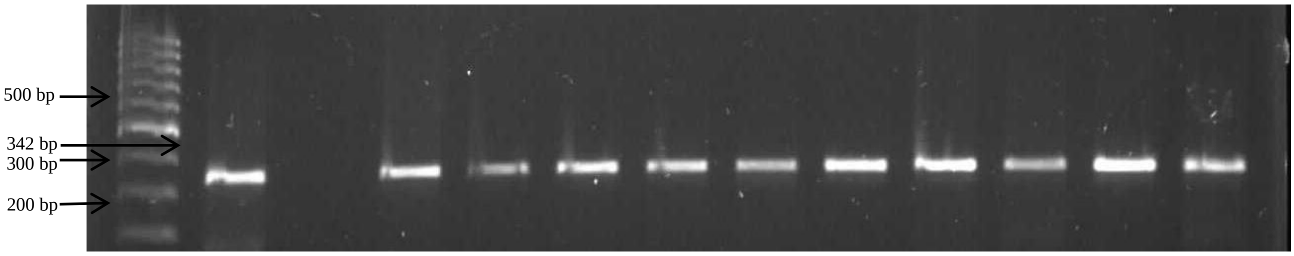

Supplementary Figure 2a. Molecular detection of NMEC pathotype by the amplification of *ibe*A gene (342 bp). Lane M: 100 bp molecular weight marker (Thermo Scientific Inc.); lane P: positive control (*E. coli* DSM 10819 strain); lane N: negative control; lanes 1 to 10 *E. coli* isolates.
