# Supplementary material for: Identification and antimicrobial resistance prevalence of pathogenic Escherichia coli strains from treated wastewater effluents in Eastern Cape, South Africa
Source: Microbiologyopen. 2016 Jan 13;5(1):143–51. doi: 10.1002/mbo3.319 (PMC4767426; doi:10.1002/mbo3.319)
Supplement: Supplementary file 5 — Figure S2b. Molecular detection of UPEC pathotype by the amplification of papC gene (382 bp). Lane M: 100 bp molecular weight marker (Thermo Scientific Inc.); lane P: positive control (E. coli DSM 4816 strain); lane N: negative control; lanes 1 to 10 E. coli isolates. [file MBO3-5-143-s005.pdf]

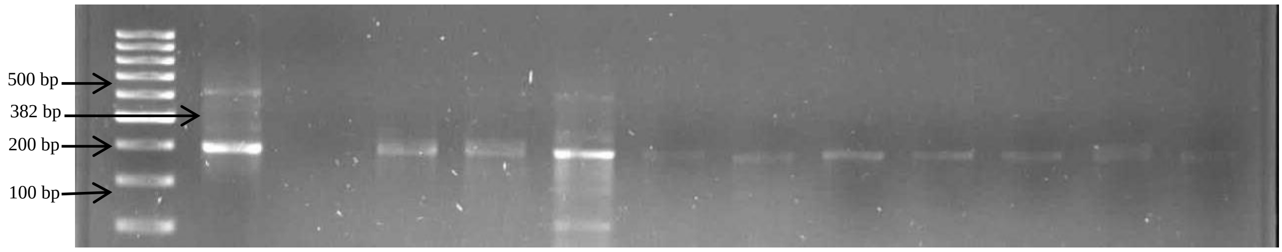

Supplementary Figure 2b. Molecular detection of UPEC pathotype by the amplification of *pap*C gene (382 bp). Lane M: 100 bp molecular weight marker (Thermo Scientific Inc.); lane P: positive control (*E. coli* DSM 4816 strain); lane N: negative control; lanes 1 to 10 *E. coli* isolates.
