# Supplementary material for: Identification and antimicrobial resistance prevalence of pathogenic Escherichia coli strains from treated wastewater effluents in Eastern Cape, South Africa
Source: Microbiologyopen. 2016 Jan 13;5(1):143–51. doi: 10.1002/mbo3.319 (PMC4767426; doi:10.1002/mbo3.319)
Supplement: Supplementary file 8 — Figure S2e. Molecular detection of EPEC pathotype by the amplification of eae gene (482 bp). Lanes M & Z: 100 bp molecular weight marker (Thermo Scientific Inc.); lane P: positive control (E. coli DSM 10973 strain); lane N: negative control; lanes 1 to 19 E. coli isolates. [file MBO3-5-143-s008.pdf]

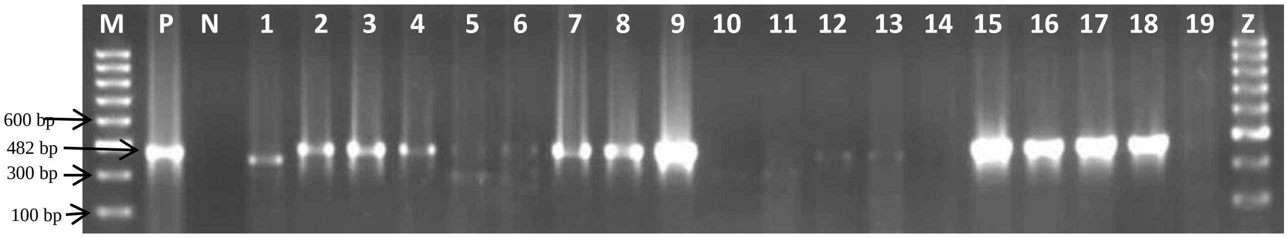

Supplementary Figure 2e. Molecular detection of EPEC pathotype by the amplification of *eae* gene (482 bp). Lanes M & Z: 100 bp molecular weight marker (Thermo Scientific Inc.); lane P: positive control (*E. coli* DSM 10973 strain); lane N: negative control; lanes 1 to 19 *E. coli* isolates.
